# Inflammation in Idiopathic Intracranial Hypertension: An Immunometabolic Mechanistic Framework and Clinical Implications

**DOI:** 10.1002/cns.70827

**Published:** 2026-03-10

**Authors:** Guangyu Han, Jiahao Song, Shuling Wan, Xunming Ji, Da Zhou, Ran Meng

**Affiliations:** ^1^ Department of Neurology Xuanwu Hospital, Capital Medical University Beijing China; ^2^ Advanced Center of Stroke Beijing Institute for Brain Disorders Beijing China; ^3^ National Center for Neurological Disorders Xuanwu Hospital, Capital Medical University Beijing China; ^4^ Capital Medical University Beijing China

**Keywords:** biomarkers, cerebrospinal fluid dynamics, idiopathic intracranial hypertension, immunometabolic axis, inflammation, targeted therapy

## Abstract

**Background:**

Idiopathic intracranial hypertension (IIH) is a multifactorial disorder characterized by sustained intracranial pressure (ICP) elevation in the absence of identifiable causes, predominantly affecting obese women of reproductive age. Although the pathophysiology of IIH remains incompletely understood, accumulating evidence indicates that inflammation is closely intertwined with metabolic, vascular, and cerebrospinal fluid (CSF) disturbances.

**Methods:**

We performed a structured literature search of PubMed, EMBASE, Web of Science, and the Cochrane Library for studies relevant to the pathophysiology of IIH, with a particular focus on inflammatory mechanisms, molecular signatures, and clinical correlates.

**Results:**

The reviewed evidence indicates that inflammatory activation within the choroid plexus and cerebral endothelium is associated with enhanced activity of sodium‐potassium adenosine triphosphatase (Na^+^/K^+^‐ATPase), Na^+^‐K^+^‐2Cl^−^ cotransporter (NKCC1), and aquaporin‐1 (AQP1), impaired barrier integrity, and increased CSF secretion. In parallel, inflammatory fibrosis of arachnoid villi and dysfunction of glymphatic–lymphatic outflow pathways may impede CSF reabsorption, further contributing to ICP elevation. At the systemic level, obesity‐associated adipokines, proinflammatory cytokines, and endocrine dysregulation—particularly involving glucocorticoid and sex‐steroid signaling—appear to amplify neuroinflammatory cascades linked to altered CSF homeostasis. Clinically, patients with IIH exhibit elevated inflammatory biomarkers (C‐reactive protein, neuron‐specific enolase, neutrophil‐to‐lymphocyte and platelet‐to‐lymphocyte ratios), intrathecal immunoglobulin G (IgG) synthesis, and increased neurofilament light chain levels, consistent with combined immune activation and neuroaxonal stress. Common comorbidities such as anemia, obstructive sleep apnea, and thrombophilia may further exacerbate inflammatory and hypoxic burden. Collectively, these findings support a conceptual shift from IIH as a purely mechanical disorder toward an immunometabolic disease of CSF regulation.

**Conclusions:**

In this review, we integrate mechanistic, clinical, and molecular evidence linking inflammation to IIH pathophysiology, and discuss how inflammatory biomarkers, metabolic modulators, and targeted anti‐inflammatory strategies could inform future diagnostic, prognostic, and therapeutic frameworks. A more precise understanding of these immunometabolic pathways may help redefine IIH as a biologically stratified and therapeutically tractable disorder.

## Graphical abstract

Systemic metabolic dysfunction and comorbidities may act as upstream modulators converging on inflammatory activation, which is closely intertwined with excessive CSF production and impaired clearance, potentially contributing to sustained ICP elevation. Targeting inflammatory pathways may offer novel therapeutic opportunities, while biomarker‐based and phenotype‐aware precision strategies could enable earlier diagnosis, individualized treatment, and dynamic monitoring of therapeutic response.

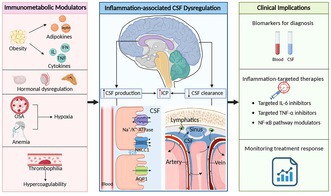

**Abbreviations**
11β‐HSD1: 11β‐hydroxysteroid dehydrogenase type 1
AQP: aquaporin
BBB: blood–brain barrier
CCL: C‐C motif chemokine ligand
CNS: central nervous system
CRP: C‐reactive protein
CSF: cerebrospinal fluid
GFAP: glial fibrillary acidic protein
HIF‐1α: hypoxia‐inducible factor‐1α
ICP: intracranial pressure
IFN‐γ: interferon‐γ
IgG: immunoglobulin G
IIH: idiopathic intracranial hypertension
IL: interleukin
MMP: matrix metalloproteinase
Na^+^/K^+^‐ATPase: sodium‐potassium adenosine triphosphatase
NBCe2: electrogenic Na^+^‐HCO_3_^−^ cotransporter 2
NfL: neurofilament light chain
NF‐κB: nuclear factor κB
NKCC1: Na^+^‐K^+^‐2Cl^−^ cotransporter
NLR: neutrophil‐to‐lymphocyte ratio
NO: nitric oxide
NSE: neuron‐specific enolase
OCBs: oligoclonal bands
OSA: obstructive sleep apnea
PLR: platelet‐to‐lymphocyte ratio
TGF‐β: transforming growth factor‐β
TNF‐α: tumor necrosis factor‐α
VEGF: vascular endothelial growth factor

## 1 Introduction

Idiopathic intracranial hypertension (IIH) is a disorder characterized by sustained elevation of intracranial pressure (ICP) in the absence of secondary causes such as mass lesions, venous thrombosis, or infection, and with otherwise normal cerebrospinal fluid (CSF) composition [1, 2, 3, 4, 5, 6, 7]. Over the past decades, the incidence of IIH has risen markedly in parallel with the global obesity epidemic [1, 2, 3, 4, 5, 7, 8, 9]. Population‐based studies from the United Kingdom report an annual incidence of up to 4.69 per 100,000, underscoring IIH as an increasingly important public health concern [9]. The disease predominantly affects obese women of childbearing age—who carry a 4.3–15‐fold higher risk than men—and represents a leading cause of chronic headache and potentially irreversible visual impairment in young adults [1, 2, 3, 4, 5, 6, 7, 8, 10]. In addition to papilledema and headache, patients frequently experience nausea, neck and back pain, pulsatile tinnitus, and cognitive symptoms, collectively resulting in substantial functional disability and reduced quality of life [1, 3, 4, 5, 7, 8, 9, 10, 11].

Despite extensive clinical and experimental investigation, the pathogenesis of IIH remains incompletely understood. Classical models have emphasized disturbances in CSF dynamics, including increased CSF production, impaired CSF absorption, cerebral venous hypertension, and abnormalities of lymphatic or glymphatic clearance [1, 2, 3, 5, 6]. Metabolic dysregulation and endocrine perturbations have also been implicated, given the striking associations with obesity, weight gain, and female sex [4, 6, 7, 9, 12]. However, these mechanisms alone do not fully account for the marked clinical heterogeneity of IIH, its strong female and obesity predominance, or the variable response to existing therapies. Increasingly, IIH is being reconsidered not as a purely mechanical disorder of pressure regulation, but as a complex neurovascular condition shaped by systemic and central biological processes.

Within this evolving perspective, inflammation has emerged as a recurrent and cross‐cutting feature across multiple domains relevant to IIH pathophysiology. Clinical studies report elevated levels of proinflammatory cytokines and chemokines—including interleukin (IL)‐1β, IL‐2, IL‐6, tumor necrosis factor‐α (TNF‐α), interferon‐γ (IFN‐γ), and C‐C motif chemokine ligand 2 (CCL2)—in serum and/or CSF of patients with IIH compared with controls [13, 14, 15, 16, 17, 18, 19, 20]. Proteomic analyses of CSF further reveal upregulation of inflammatory and immunomodulatory proteins such as immunoglobulin heavy constant alpha 1, α₁‐antitrypsin, serotransferrin, and haptoglobin, alongside obesity‐related markers including osteopontin and fibrinogen‐γ chain [21, 22]. More recent mass spectrometry‐based studies have identified dozens of differentially expressed CSF proteins converging on inflammatory signaling, extracellular‐matrix remodeling, and endothelial dysfunction pathways [23]. Collectively, these observations suggest that inflammatory activation is closely intertwined with metabolic disturbance, venous pathology, and altered CSF homeostasis in IIH.

Importantly, current evidence does not establish inflammation as a singular or universal causal driver of IIH. Rather, available data—largely derived from observational human studies and supported by experimental models—indicate that inflammation may represent an integrative interface linking systemic metabolic stressors, endocrine abnormalities, and common comorbidities to disturbances in CSF production and clearance [13, 16, 17, 24, 25]. In this context, inflammation may contribute to the maintenance and amplification of ICP elevation and may also reflect downstream responses to sustained mechanical and metabolic stress, with its relative contribution varying across disease stage and clinical phenotype.

**FIGURE 1:**
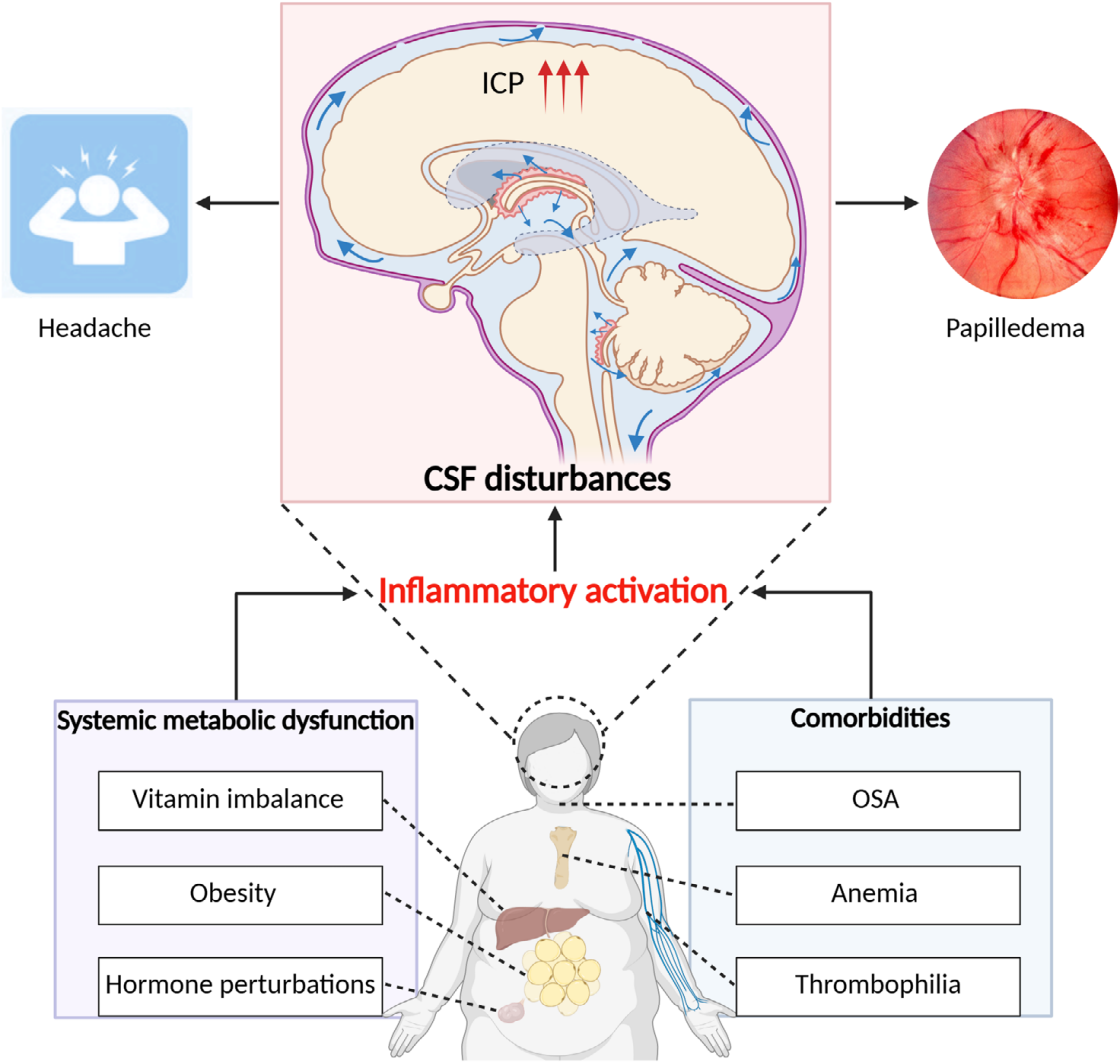
Inflammation‐centered conceptual framework of IIH. Systemic metabolic dysfunction and comorbidities may act as upstream modulators that converge on inflammatory activation, which occupies a central position within a conceptual framework linking these factors to disturbances in CSF homeostasis. Inflammatory processes are associated with excessive CSF production and impaired CSF reabsorption, potentially contributing to sustained ICP elevation and characteristic clinical manifestations, including headache and papilledema. CSF, cerebrospinal fluid; ICP, intracranial pressure; IIH, idiopathic intracranial hypertension; OSA, obstructive sleep apnea.

In this review, we synthesize clinical, molecular, and experimental evidence to reposition IIH as an immunometabolic disorder of CSF regulation. We propose an integrative conceptual framework in which inflammatory activation connects systemic metabolic dysfunction, endocrine perturbations, venous and lymphatic abnormalities, and CSF dysregulation (Figure 1). To support this framework, we performed a structured literature search of PubMed, EMBASE, Web of Science, and the Cochrane Library from database inception to November 2025, limited to publications in English. Search terms included combinations of “idiopathic intracranial hypertension”, “pseudotumor cerebri”, “benign intracranial hypertension”, “pathophysiology”, “inflammation”, “inflammatory markers”, and related keywords. We prioritized human observational and interventional studies where available, complemented by mechanistic animal and cellular studies that directly informed the proposed pathways. Literature primarily addressing secondary intracranial hypertension with identifiable causes (e.g., cerebral venous thrombosis, mass lesions, or infection) was not the focus of this review. This approach allowed us to focus on inflammation‐related mechanisms most directly relevant to the pathophysiology and clinical spectrum of IIH.

## 2 Inflammation and CSF Dysregulation

Dysregulation of CSF dynamics—through increased production and/or impaired reabsorption—remains central to prevailing models of IIH pathogenesis [1, 2, 3, 4, 6, 9, 10, 11, 26, 27]. Accumulating evidence indicates that inflammatory signaling is associated with disturbances of CSF homeostasis across multiple anatomical and physiological compartments, with potential effects on both CSF generation and clearance that are linked to ICP elevation [4, 26, 28, 29, 30, 31, 32]. Importantly, the evidentiary strength differs substantially across pathways, with many mechanistic links supported primarily by experimental models and indirect clinical correlates. In the sections below, we explicitly distinguish human data from model‐based inference and highlight clinically testable validation avenues.

### 2.1 Inflammation and CSF Hypersecretion

Most CSF is produced by the choroid plexus epithelium, where polarized apical transporters—including sodium‐potassium adenosine triphosphatase (Na^+^/K^+^‐ATPase), Na^+^‐K^+^‐2Cl^−^ cotransporter (NKCC1), Na^+^‐HCO_3_^−^ cotransporter (such as NBCe2), and aquaporin‐1 (AQP1)—drive active ion and water flux from blood into the ventricular system [2, 3, 4, 6, 9, 24, 26, 31, 33]. A smaller fraction of CSF derives indirectly from interstitial fluid generated by transcapillary exchange across the blood–brain barrier (BBB) [2, 3, 6, 33].

**FIGURE 2:**
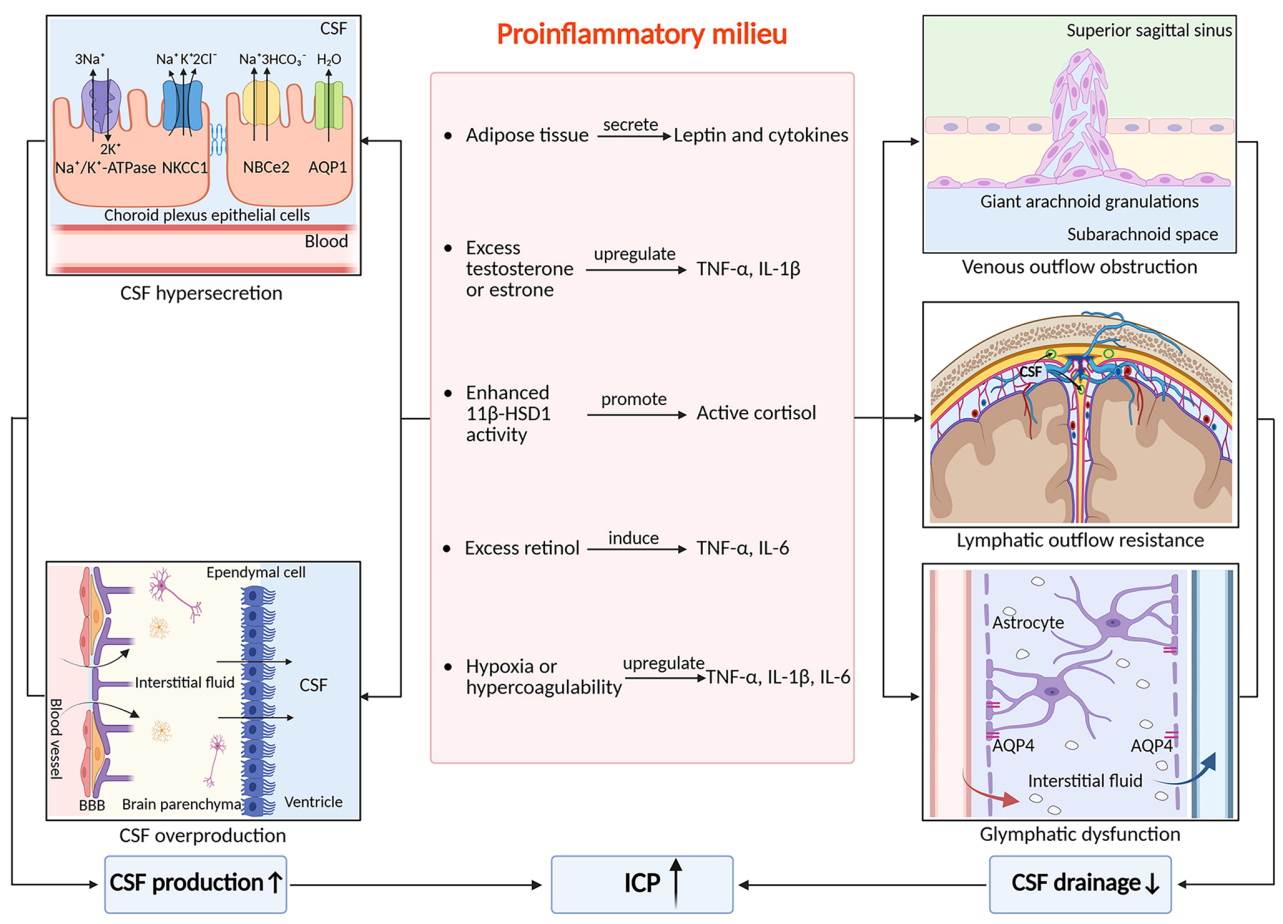
Inflammatory mechanistic framework linking systemic metabolic dysfunction and comorbidities to CSF dysregulation in IIH. Systemic metabolic dysfunction and prevalent comorbidities converge to establish a proinflammatory milieu in IIH. Adipose tissue‐derived inflammation contributes through increased secretion of leptin and proinflammatory cytokines. Dysregulated hormonal axes—including androgen excess, estrogen elevation, and glucocorticoid overactivation via 11β‐HSD1—are associated with amplified inflammatory signaling, while vitamin A dysregulation may further potentiate cytokine responses. Concurrently, comorbidities such as anemia, OSA, and thrombophilia may exacerbate the inflammatory burden through hypoxia‐ and hypercoagulability‐driven pathways. Within this proinflammatory milieu, cytokines and adipokines act on multiple compartments regulating CSF homeostasis. Proinflammatory mediators—including IL‐1β, IL‐6, TNF‐α, and leptin—have been implicated in enhanced Na^+^/K^+^‐ATPase and NKCC1 activity and upregulated AQP1 expression in the choroid plexus, together with BBB dysfunction, potentially favoring CSF hypersecretion. IL‐6 and TGF‐β are associated with inflammatory fibrosis of arachnoid villi and increased venous outflow resistance. In parallel, endothelial injury caused by TNF‐α and IL‐6, perivascular inflammation, and AQP4 mislocalization at astrocytic endfeet may impair glymphatic–lymphatic clearance. Collectively, these inflammatory processes are linked to increased CSF production, reduced CSF drainage, and sustained ICP elevation in IIH. 11β‐HSD1, 11β‐hydroxysteroid dehydrogenase type 1; AQP, aquaporin; BBB, blood–brain barrier; CSF, cerebrospinal fluid; ICP, intracranial pressure; IIH, idiopathic intracranial hypertension; IL, interleukin; Na^+^/K^+^‐ATPase, sodium‐potassium adenosine triphosphatase; NBCe2, electrogenic Na^+^‐HCO_3_^−^ cotransporter 2; NKCC1, Na^+^‐K^+^‐2Cl^−^ cotransporter; OSA, obstructive sleep apnea; TGF‐β, transforming growth factor‐β; TNF‐α, tumor necrosis factor‐α.

Direct human evidence for transporter upregulation in IIH remains limited; current support derives predominantly from experimental systems and indirect clinical associations. Within these constraints, inflammatory signaling has been implicated in CSF hypersecretion through several convergent mechanisms. In experimental models, proinflammatory cytokines enhance Na^+^/K^+^‐ATPase and NKCC1 activity and increase AQP1 expression, consistent with a choroid plexus hypersecretory phenotype [24, 25, 26, 32, 34]. In parallel, inflammation‐associated endothelial dysfunction has been linked to compromised BBB disruption and increased vascular permeability, potentially facilitating fluid transudation and CSF volume loading (Figure 2) [30, 35, 36, 37, 38]. Together, these findings support a biologically plausible “choroid plexus–endothelium inflammatory axis”. However, confirmation in well‐phenotyped IIH cohorts is still required, ideally using transport‐relevant human surrogates such as paired serum–CSF profiling, physiological secretion indices, or longitudinal pre‐ and post‐intervention trajectories.

### 2.2 Inflammation and Impaired CSF Reabsorption

Under physiological conditions, CSF is reabsorbed predominantly through arachnoid villi and granulations into the dural venous sinuses, driven by the transcompartmental pressure gradient [2, 3, 11, 24, 26, 31]. Complementary outflow routes include meningeal lymphatic vessels and the glymphatic system, which channel CSF toward cervical lymphatics and venous drainage pathways [2, 3, 4, 33, 39, 40]. Disruption of any component—via structural obstruction, venous hypertension, or lymphatic dysfunction—increases resistance to CSF outflow and contributes to ICP elevation [2, 3, 4, 6, 8, 10, 27, 31, 41]. Inflammatory processes have been associated with impairment at each level, through both structural remodeling and biochemical perturbation of barrier and perivascular transport mechanisms [25, 28, 29, 30, 31, 35, 38, 42].

#### 2.2.1 Arachnoid Granulation Dysfunction and Inflammation

Inflammation can induce fibrotic remodeling and adhesions within the arachnoid villi, which may impede CSF passage and enlarge granulations. Hypertrophic or “giant” arachnoid granulations may protrude into the dural sinus lumen, causing focal venous sinus stenosis and increasing CSF outflow resistance (Figure 2) [8, 28, 29, 43, 44, 45]. Histopathological observations suggest that inflammatory fibrosis may render villi functionally inefficient and further constrain reabsorption capacity [28, 43, 45]. While these findings provide human tissue‐level support for an inflammatory‐fibrotic substrate of impaired CSF egress, prospective studies linking granulation morphology to inflammatory profiles and longitudinal clinical outcomes remain lacking.

#### 2.2.2 Cerebral Venous Hypertension and Inflammation

Cerebral venous hypertension is a key hemodynamic determinant of elevated ICP in IIH [2, 4, 8, 11]. Bilateral transverse sinus stenoses are observed in most patients, producing venous congestion and increased sinus pressure that raises resistance to CSF outflow [1, 2, 3, 5, 6, 7, 8, 10, 11, 26, 41, 46]. Venous sinus stenting can reverse these hemodynamic abnormalities and lower ICP. This observation highlights a key pathophysiological link between venous obstruction and CSF retention [1, 2, 3, 5, 6, 7, 8, 9, 11, 26, 47]. Beyond sinus‐level narrowing, abnormalities of internal jugular venous valves have also been described, suggesting that impaired extracranial venous drainage may further exacerbate intracranial venous pressure [48, 49].

Inflammation has been proposed as a contributor to venous and valvular remodeling in IIH [42, 48, 50, 51, 52, 53, 54, 55, 56, 57]. However, the supporting evidence is derived largely from observational studies and extrapolation from other venous pathologies. In this context, proinflammatory cytokines—including IL‐6, IL‐8, and transforming growth factor‐β (TGF‐β)—together with matrix metalloproteinases (MMP‐2 and MMP‐9) are upregulated in incompetent venous valves and stenotic lesions. These mediators implicate inflammatory signaling in extracellular‐matrix remodeling and structural venous alteration [48, 53, 54, 55]. Systemic inflammatory states are further associated with endothelial injury, oxidative stress, and venous wall thickening, promoting leukocyte recruitment, fibrosis, and intimal hyperplasia that plausibly increase venous resistance [42, 50, 51, 52, 53, 54, 55]. Conversely, reverse causality is biologically plausible: elevated ICP and mechanical venous narrowing may alter shear stress and endothelial function, secondarily amplifying inflammatory activation [11, 26, 41, 42, 54, 56, 57]. These observations support a bidirectional feedback model in which venous hypertension and inflammation may reinforce one another. Longitudinal studies integrating venous hemodynamics, vessel‐wall imaging, and systemic–CSF inflammatory profiling will be essential to clarify directionality and quantify inflammatory contributions to venous remodeling in IIH.

#### 2.2.3 Lymphatic and Glymphatic Pathway Impairment and Inflammation

The meningeal lymphatic and glymphatic systems play central roles in CSF clearance and interstitial waste removal [4, 30, 31, 38, 40, 58, 59]. Impairment of these pathways has been consistently observed in patients with IIH and is associated with reduced CSF reabsorption and elevated ICP [30, 31, 60]. Neuroimaging studies further demonstrate diminished glymphatic flow and tracer clearance across multiple brain regions, linking perivascular transport failure to symptom burden [38, 40, 58, 59].

Inflammatory signaling may disrupt this clearance network at several levels. Proinflammatory mediators can compromise lymphatic endothelial integrity, reducing effective permeability and impairing CSF outflow [61, 62, 63]. Cytokine‐associated BBB tight‐junction disruption permits immune mediators to penetrate the glia‐neurovascular interface, where they may induce structural disorganization and metabolic stress [4, 30, 31, 35, 36, 37, 38, 64, 65, 66, 67]. These changes can damage astrocytic endfeet and disturb aquaporin‐4 (AQP4) polarity, a key determinant of perivascular water transport (Figure 2) [4, 38, 64, 68]. Across studies, AQP4 findings are heterogeneous: some report compensatory upregulation, whereas others describe reduced CSF‐to‐plasma AQP4 ratios, suggesting region‐ and stage‐dependent effects shaped by local inflammation and methodological variability [6, 24, 31, 32, 38, 64, 68, 69, 70, 71, 72]. Experimental data indicate that both overexpression and loss of AQP4 can perturb ICP homeostasis, and AQP4 deficiency in transgenic mouse models elevates ICP and produces an IIH‐like phenotype [73, 74, 75, 76].

Astrocyte reactivity provides an additional mechanistic layer [38, 71, 77]. Biopsy findings and CSF biomarkers demonstrate patchy astrogliosis and increased glial fibrillary acidic protein (GFAP) levels in IIH, consistent with astrocyte activation [36, 37, 71, 78]. Neuroinflammatory and autoimmune processes—including anti‐GFAP autoantibodies—may directly damage astrocytes and obstruct glymphatic flow [36, 38, 77, 79]. Inflammatory cytokines can further impair mitochondrial metabolism in perivascular astrocytes, exacerbating energy failure and fluid stasis [3].

Collectively, these observations support a “glymphatic–astroglial inflammatory axis,” linking BBB leakage, AQP4 polarity disruption, and astrocytic injury to impaired glymphatic–lymphatic clearance and sustained intracranial hypertension. This framework is supported by converging evidence from human neuroimaging and biomarker studies, complemented by mechanistic insights from animal models.

## 3 Inflammation and Systemic Metabolic Dysfunction

Building on evidence that inflammatory signaling intersects with CSF homeostasis, recent work has prompted a reconceptualization of IIH as an immunometabolic disorder [1, 2, 4, 5, 8, 12]. Obesity, weight gain, and hormonal dysregulation appear to create a systemic proinflammatory milieu that may amplify ICP elevation [14, 15, 80, 81, 82, 83, 84, 85]. Chronic metabolic inflammation has been linked to disturbances in both CSF production and clearance, situating IIH within a broader spectrum of immunometabolic diseases. Importantly, most available human data are cross‐sectional and potentially confounded; mechanistic interpretations should therefore be regarded as association‐based and hypothesis‐generating unless supported by longitudinal or interventional evidence.

### 3.1 Obesity, Inflammatory Mediators, and IIH

Obesity is the strongest modifiable risk factor for IIH and closely parallels its rising global incidence [1, 2, 4, 6, 8, 9, 41, 80, 86, 87, 88, 89]. Adipose tissue functions as an endocrine and immune‐active organ, secreting adipokines and cytokines—including leptin, IL‐1β, IL‐6, TNF‐α, IFN‐γ, and various chemokines—that sustain a state of chronic low‐grade inflammation [2, 5, 6, 9, 14, 15, 16, 19, 26, 35, 41, 80, 81, 86, 87, 88, 89, 90]. Multiple studies report abnormal levels of these mediators in serum and/or CSF from patients with IIH, supporting an association between adipose‐derived inflammation and disease phenotype [2, 6, 14, 15, 16, 19, 26, 35, 41, 80, 81, 87, 88, 89, 90, 91].

When stratified by biological compartment, disease activity, and assay modality, a reproducible—yet methodologically variable—pattern emerges (Table S1). Several cohorts demonstrate higher serum IL‐1β and TNF‐α and elevated CSF IL‐2 and IL‐6 relative to controls, whereas smaller or remission‐phase studies often report null findings [15, 16, 17, 18, 19, 79, 92, 93]. Findings for IL‐12 and IFN‐γ remain inconsistent [13, 16, 17]. By contrast, evidence implicating the Th17 axis is more coherent: CSF IL‐17 and IL‐23 are frequently increased, whereas the anti‐inflammatory IL‐10 is often reduced, suggesting a Th17‐skewed intrathecal immune milieu [13, 16, 17, 18, 93]. Chemokine profiles align with this pattern, with CCL2 commonly elevated in CSF and CCL7/CCL8 in serum, indicating augmented monocyte and lymphocyte recruitment [14, 94]. Such heterogeneity likely reflects differences in disease activity, matching criteria (age, sex, body mass index), biospecimen type (serum vs. CSF), and assay platform (multiplex vs. enzyme‐linked immunosorbent assay [ELISA]). Future studies should prioritize standardized designs and paired serum–CSF sampling to improve interpretability.

Mechanistically, this inflammatory profile is compatible with choroid‐plexus and cerebral endothelial activation, processes that may influence ion transport, barrier integrity, and CSF homeostasis (see Sections 2.1 and 2.2). Leptin is among the most consistently elevated signals in both serum and CSF in IIH, whereas adiponectin generally trends lower, albeit variably [2, 4, 5, 6, 14, 19, 26, 30, 34, 35, 95, 96, 97, 98, 99, 100]. These adipokines, together with cytokines and chemokines, constitute an interlinked network coupling metabolic inflammation to altered CSF dynamics (Figure 2). Experimental studies indicate that inflammatory signaling can promote endothelial dysfunction, disrupt the BBB, and enhance Na^+^/K^+^‐ATPase and NKCC1 activity in choroid‐plexus epithelia—mechanisms linked to increased CSF secretion and outflow resistance [25, 30, 79, 86, 89, 94, 99, 101, 102, 103, 104, 105, 106]. In rodent models, TNF‐α increases CSF production and CCL2 impedes drainage, both contributing to ICP elevation [25]. Clinically, serum TNF‐α inversely correlates with visual‐field grade, and higher IL‐1β or IL‐8 predicts relapse, linking cytokine dysregulation to disease activity [15, 79]. Together, these observations support an “obesity–inflammation–CSF” axis, while underscoring the need for longitudinal, phenotype‐stratified human studies to distinguish initiating drivers from secondary inflammatory signatures.

### 3.2 Hormonal Perturbations, Inflammatory Activation, and IIH

Beyond adipose‐derived inflammation, hormonal dysregulation—particularly involving sex steroids and glucocorticoids—has emerged as an important modulator of immune signaling and CSF imbalance in IIH [1, 2, 4, 6, 8, 9, 26]. These endocrine pathways interact bidirectionally with inflammatory cascades and may influence endothelial function, BBB permeability, and choroid plexus transport in ways associated with elevated ICP [35, 84, 107, 108, 109, 110]. Evidence in humans is predominantly observational, supported by mechanistic data from experimental models.

Sex hormone alterations are among the most reproducible metabolic abnormalities in IIH. Serum and CSF concentrations of testosterone and estrone are elevated in affected women compared with matched controls, whereas androgen profiles in men with IIH are more heterogeneous [2, 3, 4, 9, 107, 111, 112, 113, 114]. Excess androgens and estrogens can potentiate endothelial inflammation and oxidative stress, plausibly impairing BBB integrity and cerebrovascular autoregulation (Figure 2) [24, 82, 83, 84, 108, 109, 115]. Experimental studies further show that testosterone and estrone upregulate TNF‐α and IL‐1β, which in turn enhance Na^+^/K^+^‐ATPase and NKCC1 activity within the choroid plexus and increase CSF secretory flux [1, 2, 3, 4, 9, 25, 41, 84, 107, 109, 112, 113, 116, 117]. These findings are consistent with a “sex hormone–inflammation–CSF” axis that may contribute to the female predominance and hormonally sensitive phenotype of IIH, although direct causal validation in humans is lacking.

Dysregulation of glucocorticoid metabolism may further amplify inflammatory signaling [2, 3, 4, 9, 24, 86]. The enzyme 11β‐hydroxysteroid dehydrogenase type 1 (11β‐HSD1), abundantly expressed in the choroid plexus and arachnoid granulations, converts inactive cortisone to active cortisol and regulates local glucocorticoid tone [2, 4, 9, 25, 86, 99, 118, 119, 120]. Elevated 11β‐HSD1 activity has been reported in IIH and enhanced by TNF‐α, IL‐1β, IL‐6, and leptin [25, 79, 85, 86]. Increased local cortisol regeneration is associated with augmented Na^+^/K^+^‐ATPase activity and morphological remodeling of arachnoid granulations, linking glucocorticoid signaling to both CSF hypersecretion and increased outflow resistance (Figure 2) [2, 3, 9, 24, 25, 79, 86, 99, 110, 118, 119, 121]. This supports a feed‐forward endocrine–inflammatory loop in which inflammation and glucocorticoid signaling may mutually reinforce CSF dysregulation.

Additional endocrine axes may intersect with inflammatory pathways. CSF proteomic studies report reduced angiotensinogen and angiotensin II in IIH, potentially affecting cerebrovascular tone and BBB integrity [21, 122, 123, 124]. Experimental models show that angiotensinogen deficiency impairs tight‐junction organization, particularly under inflammatory stress, while attenuation of the angiotensin‐converting enzyme 2 (ACE2)/angiotensin‐(1–7)/Mas receptor axis diminishes anti‐inflammatory and vasoprotective signaling, favoring microvascular leakage and vasogenic edema [125, 126, 127, 128].

The natriuretic peptide system likewise integrates into this hormonal‐inflammatory network. Plasma concentrations of C‐type natriuretic pro‐peptide (pro‐CNP) are significantly reduced in patients with IIH compared with controls, consistent with endothelial dysfunction and impaired vasoregulatory capacity [6, 24, 26, 129, 130]. Such deficiency may attenuate nitric oxide (NO)‐mediated vasorelaxation and thereby favor inflammatory activation and cerebral perfusion dysregulation, processes plausibly linked to sustained intracranial hypertension [6, 26, 129].

### 3.3 Dysregulated Vitamin Metabolism, Inflammatory Responses, and IIH

Dysregulated vitamin A metabolism has long been implicated as a potential contributor to intracranial hypertension [6, 131, 132, 133]. Comparative studies in IIH versus matched controls have evaluated multiple vitamin A derivatives, with several reporting elevated serum and CSF retinol concentrations, suggesting a possible role for hypervitaminosis A‐related pathways in IIH phenotypes [5, 6, 24, 26, 32, 97, 131, 133, 134, 135]. However, human evidence remains heterogeneous and occasionally conflicting.

Mechanistically, excess retinol bound to retinol‐binding protein (RBP) can activate nuclear factor κB (NF‐κB) signaling, which induces TNF‐α and IL‐6 release from endothelial and immune cells [136, 137, 138, 139]. These cytokines, in turn, have been shown experimentally to upregulate AQP1 expression in the choroid plexus and increase CSF secretion. Concomitantly, inflammatory stress may compromise endothelial junctions and barrier integrity, leading to vasogenic edema and ICP elevation [32, 34, 132]. Inflammatory injury to arachnoid granulations may further impair cellular integrity and hinder CSF reabsorption, thereby potentially reinforcing the cycle of intracranial hypertension (Figure 2) [5, 24, 70, 133, 134, 135].

The association between vitamin A metabolism and IIH remains debated. Several clinical studies report no significant differences in serum or CSF vitamin A derivatives between IIH and controls [34, 132, 134]. These inconsistencies likely reflect heterogeneity in analytical methods, dietary intake, and case–control matching criteria. Interestingly, experimental models suggest an opposing pathological pattern: vitamin A deficiency in rats and calves induces arachnoid villi fibrosis, impaired CSF reabsorption, and subsequent ICP elevation [24, 70, 131]. This paradox raises the possibility that both excess and deficiency of vitamin A may perturb CSF dynamics through distinct yet convergent mechanisms—the former predominantly via inflammatory activation and barrier injury, and the latter through structural fibrosis and reabsorptive failure. Overall, the pathway is primarily supported by heterogeneous human observational data and biologically plausible animal models, underscoring the need for standardized metabolic profiling and integrated serum–CSF analyses in prospective, well‐characterized IIH cohorts.

Taken together, systemic metabolic disturbances—including adipokine imbalance, hormonal dysregulation, and micronutrient disruption—appear to converge on shared inflammatory pathways that modulate Na^+^/K^+^‐ATPase activity, AQP1 expression, endothelial permeability, and arachnoid function. These inflammation‐related perturbations may alter CSF production and clearance and thereby contribute to sustained ICP elevation in IIH.

## 4 Clinical and Molecular Evidence of Inflammatory Involvement in IIH

### 4.1 Elevated Inflammatory Biomarkers

Multiple peripheral inflammatory biomarkers—including the neutrophil‐to‐lymphocyte ratio (NLR), platelet‐to‐lymphocyte ratio (PLR), neuron‐specific enolase (NSE), and C‐reactive protein (CRP)—are reported to be elevated in patients with IIH compared with controls [106, 140, 141, 142]. These alterations broadly reflect systemic immune activation and have been linked to BBB dysfunction and impaired CSF outflow pathways associated with intracranial hypertension [35, 142, 143, 144]. Clinically, higher NLR and PLR correlate with best‐corrected visual acuity, retinal nerve‐fiber‐layer thickness, and papilledema grade, supporting an association between systemic inflammation and visual dysfunction [106, 140]. Consistent with these peripheral findings, CSF proteomic analyses demonstrate increased levels of lipocalin‐2, sortilin‐1, and autotaxin, alongside decreased decorin, an anti‐inflammatory mediator [89]. Collectively, these biomarker profiles support a shift toward a proinflammatory systemic and intrathecal milieu in IIH. However, most markers are nonspecific and susceptible to confounding by obesity and related comorbidities, underscoring the need for phenotype‐aware interpretation and longitudinal validation before clinical application.

### 4.2 Immune Dysregulation

Beyond innate immune activation, emerging evidence highlights involvement of adaptive immune pathways in IIH [6, 16, 64, 145, 146, 147]. Oligoclonal bands (OCBs) are detected in approximately 30% of patients, often accompanied by intrathecal immunoglobulin G (IgG) synthesis and elevated serum globulin levels [16, 20, 72, 140, 145, 146, 147]. Additional studies describe heterogeneous IgG binding patterns and T‐cell‐mediated immune activity in CSF, consistent with chronic intrathecal immune activation [6, 64, 72, 147]. Notably, OCB positivity and elevated serum globulin concentrations have been associated with more severe visual impairment and increased recurrence risk, suggesting that adaptive immune activation may characterize a more persistent or relapsing disease course [16, 140]. At the same time, OCBs are not specific to IIH and necessitate careful exclusion of alternative inflammatory central nervous system (CNS) disorders in both clinical practice and research cohorts. Future studies should clarify whether OCB‐positive IIH represents a reproducible immunological endophenotype with distinct prognostic, biomarker, or therapeutic implications.

### 4.3 Increased Neurofilament Light Chain Levels

Neurofilament light chain (NfL), a sensitive marker of axonal injury, provides additional molecular evidence of neuroaxonal involvement in IIH and has been widely studied across inflammatory neurological diseases [148, 149]. Multiple case–control studies demonstrate elevated CSF NfL, often accompanied by increased plasma concentrations and higher CSF‐to‐plasma ratios [78, 148, 150]. Higher NfL levels correlate with severe papilledema and visual‐field defects, consistent with optic nerve vulnerability in advanced disease [150, 151]. A raised CSF‐to‐plasma NfL ratio has been proposed as a surrogate marker of impaired glymphatic clearance and altered CSF dynamics, although this interpretation remains hypothesis‐generating [78, 148, 150].

Overall, NfL appears to be a clinically informative marker of neuroaxonal injury in IIH. Longitudinal studies are required to determine whether NfL reflects upstream inflammatory activity, downstream mechanical injury, or an interaction between the two.

### 4.4 Genetic and Microbiome Evidence

Emerging molecular profiling approaches further expand the inflammatory landscape of IIH. Exosomal transcriptome analyses from CSF and plasma reveal upregulation of inflammation‐related genes and IL‐mediated signaling pathways, indicating coordinated immune activation across central and peripheral compartments [91]. Complementary metagenomic studies of the gut microbiome report a reduced abundance of *Lactobacillus ruminis* ( *L. ruminis* ) in IIH relative to obese controls [152]. As *L. ruminis* can suppress NF‐κB activation and IL‐8 production, its depletion may weaken mucosal anti‐inflammatory defenses and favor a systemic proinflammatory milieu associated with ICP dysregulation [152, 153]. Given limited sample sizes and cross‐sectional designs, these genetic and microbiome signals should be considered hypothesis‐generating. Replication in larger cohorts and mechanistic studies linking microbial, transcriptomic, and clinical outcomes will be essential to establish causality.

### 4.5 Comorbidities as Inflammatory Modulators in IIH Pathogenesis

Several comorbidities prevalent in IIH—most notably anemia, obstructive sleep apnea (OSA), and thrombophilia—may modulate inflammatory and hemodynamic pathways relevant to ICP elevation.

Anemia has been repeatedly associated with IIH, with symptom improvement reported after correction, and meta‐analyses suggest it constitutes an independent risk factor [154, 155, 156]. OSA is likewise overrepresented, and nocturnal oxygen therapy or positive airway pressure has been associated with clinical improvement in selected patients [24, 157, 158, 159, 160, 161]. Both conditions expose patients to chronic or intermittent hypoxia, promoting reactive‐oxygen‐species (ROS) formation and stabilization of hypoxia‐inducible factor‐1α (HIF‐1α) [42, 54, 162, 163, 164, 165]. The accumulation of HIF‐1α activates NF‐κB signaling, which in turn upregulates proinflammatory mediators (TNF‐α, IL‐1β, and IL‐6), and drives vascular endothelial growth factor (VEGF) overexpression [165, 166, 167, 168, 169]. Excessive NO production from inducible nitric oxide synthase (iNOS) activation contributes to cerebral vasodilation, while VEGF‐mediated tight‐junction disruption increases BBB permeability—processes culminating in elevated ICP (Figure 2) [24, 154, 155, 156, 157, 158, 159, 160, 161, 166, 167, 170, 171, 172, 173].

Thrombophilic states are also disproportionately represented in IIH, including methylenetetrahydrofolate reductase (MTHFR) C677T mutation, factor V variants, positive anticardiolipin antibodies and lupus anticoagulants, and elevated levels of factor VIII, fibrinogen, or plasminogen activator inhibitor‐1 (PAI‐1) [17, 174, 175, 176, 177, 178, 179, 180, 181, 182]. These abnormalities promote platelet activation, and CD40 ligand (CD40L) and platelet factor 4 (PF4) release, facilitating neutrophil recruitment and neutrophil extracellular traps (NETs) formation [183, 184, 185]. The resulting microvascular inflammation may damage endothelial integrity and arachnoid‐villus microcirculation, thereby reducing CSF reabsorption [17, 146, 174, 175, 176, 177, 178, 179, 180, 181]. In addition, suppression of fibrinolysis may favor fibrin deposition along arachnoid villi and amplify local inflammatory signaling [17, 174, 175, 177, 186]. Toll‐like receptor 4 (TLR4)‐mediated activation of endothelial cells and macrophages further promotes cytokine release and arachnoid fibrosis, reinforcing resistance to CSF outflow and sustaining intracranial hypertension (Figure 2) [186, 187, 188].

Beyond these comorbidities, systemic infections and chronic inflammatory disorders appear more prevalent in IIH than in obese controls [189]. This observation raises the possibility that infection‐related immune activation may act as a precipitating factor in predisposed individuals.

## 5 Inflammatory Biomarkers, Phenotypic Stratification, and Anti‐Inflammatory Therapeutic Strategies

**FIGURE 3:**
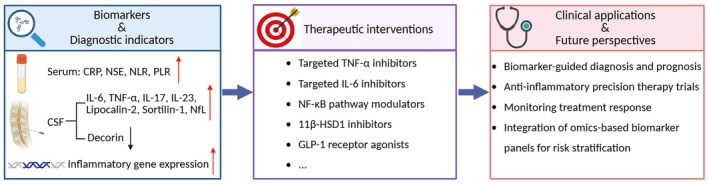
Translational framework linking inflammatory biomarkers to therapeutic targets and future perspectives in IIH. A range of inflammatory biomarkers—including elevated serum CRP, NSE, NLR, and PLR, increased CSF cytokines (IL‐6, TNF‐α, IL‐17, IL‐23), and upregulated inflammatory gene signatures in exosomes—together reflect systemic and central immune activation in IIH. Elevated NfL levels in CSF and plasma indicate neuroaxonal injury associated with disease severity, whereas reduced decorin suggests impaired anti‐inflammatory balance. From a translational perspective, these inflammatory pathways highlight potential therapeutic targets. Metabolic and anti‐inflammatory agents, including 11β‐HSD1 inhibitors, GLP‐1 receptor agonists, cytokine‐targeted biologics (e.g., TNF‐α or IL‐6 inhibitors), and modulators of NF‐κB signaling, represent emerging strategies that may complement established IIH treatments. Integration of biomarker‐informed, phenotype‐aware approaches could enable earlier diagnosis, individualized therapeutic selection, and dynamic monitoring of treatment response. 11β‐HSD1, 11β‐hydroxysteroid dehydrogenase type 1; CRP, C‐reactive protein; CSF, cerebrospinal fluid; GLP‐1, glucagon‐like peptide‐1; IIH, idiopathic intracranial hypertension; IL, interleukin; NfL, neurofilament light chain; NF‐κB, nuclear factor κB; NLR, neutrophil‐to‐lymphocyte ratio; NSE, neuron‐specific enolase; PLR, platelet‐to‐lymphocyte ratio; TNF‐α, tumor necrosis factor‐α.

Early and effective intervention is essential in IIH to prevent irreversible visual loss [1, 2, 11, 26, 158]. Current diagnostic criteria are internationally standardized and rely on clinical features, funduscopic assessment, neuroimaging, and CSF opening pressure measurement [1, 2, 3, 4, 5, 11, 26]. Contemporary management emphasizes weight reduction and carbonic‐anhydrase inhibitors, while emerging pharmacological agents—including glucagon‐like peptide‐1 (GLP‐1) receptor agonists and 11β‐HSD1 inhibitors—have shown promising efficacy in early clinical studies [1, 2, 3, 4, 5, 7, 8, 9, 10, 11, 158, 190, 191, 192]. Surgical interventions, such as CSF shunting and venous‐sinus stenting, remain reserved for refractory or vision‐threatening disease [1, 2, 3, 4, 5, 7, 8, 9, 10, 11, 191]. Within this therapeutic landscape, inflammation offers a complementary and potentially unifying framework for biomarker development, phenotypic stratification, and treatment prioritization (Figure 3).

### 5.1 Clinical‐Use Stratification of Inflammatory Biomarkers

To enhance clinical interpretability and avoid descriptive “list‐like” presentation, inflammatory biomarkers in IIH can be stratified by intended use‐case. First, risk‐ or phenotype‐supportive markers—including NLR, PLR, CRP, and NSE—are readily accessible and inexpensive but lack disease specificity and require careful adjustment for obesity and comorbid conditions [106, 140, 141, 142]. Second, severity and organ‐injury markers, most notably NfL and, where available, astroglial markers, more directly reflect neuroaxonal or optic‐nerve injury and may inform the risk of unfavorable visual outcomes [78, 148, 150]. Third, mechanistic pathway markers—such as Th17‐related cytokines, endothelial activation proteins, and CSF proteomic signatures—offer insight into dominant biological processes in individual patients and may generate testable therapeutic hypotheses [13, 16, 17, 18, 93]. Across all categories, longitudinal validation and phenotype‐aware interpretation are essential to distinguish initiating drivers from sustaining biology or downstream tissue injury and to minimize confounding by metabolic and systemic factors.

### 5.2 Phenotypic Heterogeneity and Pragmatic Stratification

IIH encompasses a heterogeneous spectrum, including typical obesity‐associated IIH, non‐obese or male IIH, fulminant presentations, IIH without papilledema, and pediatric IIH [3, 4, 5, 7, 8, 9, 10, 11]. These phenotypes likely differ in underlying drivers, inflammatory burden, and treatment responsiveness. While inflammation‐related disturbances of CSF homeostasis may represent a shared downstream pathway, metabolic and endocrine inflammatory mechanisms appear preferentially enriched in typical obesity‐related IIH. In contrast, atypical phenotypes may exhibit distinct inflammatory profiles or more context‐dependent modulatory roles [4, 26, 28, 29, 30, 84, 86].

A pragmatic stratification anchored in readily accessible clinical features—such as body mass index, sex, age, disease tempo, papilledema status, and comorbidity burden—may therefore refine interpretation of inflammatory biomarkers, guide selection of candidate markers, and support individualized management across the IIH spectrum.

### 5.3 Therapeutic Implications and Evidence‐Tier Prioritization

Corticosteroids exert potent anti‐inflammatory effects and have been reported to provide short‐term benefit in acute visual deterioration in IIH [8, 10, 24, 193, 194, 195]. However, long‐term corticosteroid therapy is discouraged due to adverse metabolic effects, including weight gain, fluid retention, and rebound intracranial hypertension after withdrawal [5, 8, 10, 24, 26, 97, 195, 196, 197, 198]. These limitations highlight the need for more targeted anti‐inflammatory strategies.

Therapeutic prioritization should be guided by evidence tier. Agents with IIH‐specific clinical signals—such as GLP‐1 receptor agonists and 11β‐HSD1 inhibitors—should remain at the forefront of disease‐modifying strategies [1, 2, 3, 4, 5, 7, 8, 9, 10, 11, 158, 190, 191, 192]. In contrast, cytokine‐targeted biologics (e.g., TNF‐α or IL‐6 inhibitors) currently remain hypothesis‐driven, supported primarily by mechanistic plausibility and limited clinical experience. Their use should therefore be confined to well‐designed clinical‐trial settings with explicit safety monitoring and careful patient selection, particularly given infection risk and the reproductive‐age female population typical of IIH. Future interventional studies incorporating phenotypic stratification and biomarker enrichment will be critical to determine whether inflammation‐modulating strategies can meaningfully improve ICP trajectories and vision‐related outcomes.

## 6 Conclusion

Accumulating clinical, molecular, and experimental evidence supports a substantive role for inflammation in the pathophysiology of IIH. Obesity, hormonal perturbations, and dysregulated vitamin metabolism may converge to generate a chronic proinflammatory milieu associated with disrupted CSF homeostasis. Adipose‐derived cytokines and adipokines, together with aberrant glucocorticoid and sex‐hormone signaling, are implicated in amplifying neuroinflammatory cascades that act on the choroid plexus, cerebral endothelium, and arachnoid villi. These processes are associated with increased activity of Na^+^/K^+^‐ATPase, NKCC1, and AQP1, impaired barrier integrity, and altered cerebrovascular tone, thereby providing biologically plausible links between inflammation, excessive CSF production, and reduced CSF reabsorption.

Parallel evidence from clinical cohorts reinforces this framework. Elevated peripheral inflammatory biomarkers, intrathecal immune activation, and increased NfL levels indicate that IIH involves both systemic and CNS‐compartment immune perturbation, bridging metabolic stress with neuroaxonal injury. Common comorbidities such as anemia, OSA, and thrombophilia may further intensify inflammatory burden through hypoxia‐ and coagulation‐driven pathways. Together, these observations support the concept of IIH as an immunometabolic disorder rather than a purely mechanical elevation of ICP. Nevertheless, most available data derive from small, observational, and heterogeneous cohorts and do not yet establish causality or clarify whether specific inflammatory signatures are disease‐initiating or disease‐sustaining.

A central priority for future research is therefore to disentangle initiating drivers—such as obesity‐associated endocrine–immune dysregulation and hypoxia‐related comorbidities—from sustaining or downstream signatures, including neuroaxonal injury and astroglial activation. Clinically, integrating inflammatory biomarkers into diagnostic and monitoring frameworks may enable earlier detection, improved risk stratification, and precision‐guided therapy, provided biomarkers are interpreted within appropriate phenotypic context and confounding is addressed. Therapeutically, targeted metabolic and immune‐modulating strategies represent a promising complement to weight‐based and surgical interventions. Progress will depend on phenotype‐stratified, biomarker‐enriched prospective cohorts; mechanistic studies dissecting compartment‐specific immune signaling; and interventional trials designed to test whether modulation of these pathways translates into durable improvements in ICP control and visual outcomes. Advancing these efforts may ultimately transform IIH from a historically idiopathic syndrome into a mechanistically defined and therapeutically tractable disorder.

## Author Contributions

D.Z. and R.M.: study conception, literature review strategy, manuscript drafting and critical revision, and final approval of the manuscript. G.H.: literature review and synthesis, manuscript drafting, and figure preparation. J.S.: literature search and figure preparation. S.W. and X.J.: literature search and data extraction. All authors read and approved the final manuscript.

## Funding

This work was supported by the National Natural Science Foundation of China (Grants 82171297 and 82101390), the Central Guidance Fund for Local Science and Technology Development (Grant ZYYD2025QY21), and the Xuanwu Hospital Elite Cultivation Program (Grant YC20250103).

## Ethics Statement

The authors have nothing to report.

## Consent

The authors have nothing to report.

## Conflicts of Interest

The authors declare no conflicts of interest.

## Supporting information


**TABLE S1:** Clinical studies of inflammatory signatures organized by functional relevance in IIH.

## Data Availability

Data sharing not applicable to this article as no datasets were generated or analyzed during the current study.
