# Supplementary material for: Inflammation in Idiopathic Intracranial Hypertension: An Immunometabolic Mechanistic Framework and Clinical Implications
Source: CNS Neurosci Ther. 2026 Mar 10;32(3):e70827. doi: 10.1002/cns.70827 (PMC12976464; doi:10.1002/cns.70827)
Supplement: Supplementary file 1 — TABLE S1: Clinical studies of inflammatory signatures organized by functional relevance in IIH. [file CNS-32-e70827-s001.docx]

**Table S1** **Clinical studies of inflammatory signatures organized by functional relevance in IIH**

| **Items** | **First author** | **IIH cases** | | **Control groups** | | | **Biological source** | **Assay methods** | **Results** | **Clinical implications** | **Limitations** |
| --- | --- | --- | --- | --- | --- | --- | --- | --- | --- | --- | --- |
|  |  | **Sample size (M/F)** | **Diagnostic criteria** | **Sample size (M/F)** | **Matching criteria** | **Cohort** |  |  |  |  |  |
| **Section I. Risk and phenotype stratification markers** | | | | | | | | | | | |
| **Leptin** | Lampl et al. 2002 | 15 (0/15) | - | 31 (0/31) | Age, sex, BMI | HC | Serum | Immunometric assay | IIH>HC (P<0.0001) | - | - |
|  | Ball et al. 2009 | 26 (0/26) | Modified Dandy | 62 (22/40) | Age, sex, BMI | Heterogeneous patients | Serum and CSF | MIA | IIH>controls (P<0.01) | - | - |
|  | Dhungana et al. 2009 | 8 | Modified Dandy | 8 | - | MS, migraine, TTH | Serum and CSF | Cytokine antibody arrays, ELISA | Serum: IIH>controls (P<0.05)  CSF: NS (P>0.05) | No correlation with CSF pressure | - |
|  | Doppler et al. 2016 | 28 (5/23) | Modified Dandy | 29 (12/17) | Age, sex | Non-IIH patients | CSF and plasma | ELISA | IIH>controls (P<0.001) | - | - |
|  | Samancı et al. 2017 | 36 (4/32) | Revised Friedman | 40 (4/36) | Age, sex, BMI | HC | Serum | MIA | NS (P=0.91) | No correlations with disease duration or visual impairment | IIH cases in remission, CSF not obtained in HC |
|  | Westgate et al. 2021 | 97 (0/97) | Modified Dandy | 43 (0/43) | Age, BMI | HC | Serum and CSF | ELISA | Serum: IIH>HC (P=0.003)  CSF: NS (P>0.05) | Neither serum nor CSF leptin levels correlated with CSF pressure | Adult women, relatively young, small sample size |
|  | Abdelghaffar et al. 2022 | 38 (0/38) | Modified Dandy | 38 (0/38) | Age | Non-IIH subjects | Serum and CSF | ELISA | IIH>controls (both P<0.001) | - | A single center study, small sample size |
| **Adiponectin** | Ball et al. 2009 | 26 (0/26) | Modified Dandy | 62 (22/40) | Age, sex, BMI | Heterogeneous patients | Serum and CSF | MIA | Serum: IIH<controls (P>0.05)  CSF: IIH<controls (P=0.0005) | - | - |
| **NLR/PLR** | Ceylan et al. 2021 | 33 (4/29) | Revised Friedman | 33 (5/28) | Age, sex, BMI | TTH | Venous blood | automated hematology analyzer | IIH>controls  IIH patients: PE>without PE (both P<0.05) | Associations with BCVA, RNFLT and PE | Retrospective design |
|  | Fahmy et al. 2024 | 36 (1/35) | ICHD-III | 36 (5/31) | Age, sex | HC | Venous blood | automated hematology analyzer | IIH>HC (P<0.05) | No correlations with headache severity, number of relapses or grade of perimetry | Small sample size |
| **CRP** | Hannerz et al. 2011 | 12 (0/12) | ICHD-II | 12 (0/12) | Age, sex | HC | Serum | - | IIH>HC (P<0.001) | Associated with CSF pressure | Small group sizes, rigorous inclusion criteria |
|  | Pollak et al. 2015 | 82 (9/73) | Modified Dandy | - | - | - | Serum | - | 51% of patients with elevated CRP, compared to normal values | Associated with unfavorable visual outcome and less improvement in visual field sensitivity | Generalizability of results |
|  | Fahmy et al. 2024 | 36 (1/35) | ICHD-III | 36 (5/31) | Age, sex | HC | Serum | - | IIH>HC (P=0.001) | Positive correlation with the presence of stenosis in MRV | Small sample size |
| **hs-CRP** | Kesler et al. 2006 | 33 (0/33) | Modified Dandy | 33 (0/33) | Age, BMI | HC | Venous blood | Nephelometry | IIH>HC (P=0.186) | - | - |
| **Fibrinogen** | Sussman et al. 1997 | 38 (4/34) | Modified Dandy | 34 | Age, BMI | HC, other neurological diseases | Plasma | Clauss method | NS (P>0.05) | - | - |
|  | Kesler et al. 2006 | 33 (0/33) | Modified Dandy | 33 (0/33) | Age, BMI | HC | Plasma | Clauss method | IIH>HC (P=0.038) | - | - |
|  | Hannerz et al. 2011 | 12 (0/12) | ICHD-II | 12 (0/12) | Age, sex | HC | Plasma | - | IIH>HC (P=0.001) | Associated with CSF pressure | Small group sizes, rigorous inclusion criteria |
| **Fibrinogen β/γ chain** | Lecube et al. 2012 | 8 (0/8) | Criteria^#^ | 8 (0/8) | Age | Non-obese IIH women | Serum and CSF | ELISA, LC-MS proteomics | Serum: NS (P=0.67)  CSF: obese IIH women>non-obese IIH women (P<0.05) | - | Selected IIH women, small sample size |
| **Osteopontin** | Lecube et al. 2012 | 8 (0/8) | Criteria^#^ | 8 (0/8) | Age | Non-obese IIH women | Serum and CSF | ELISA, LC-MS proteomics | Serum: NS (P=0.867)  CSF: Obese IIH women>non-obese IIH women (P<0.05) | - | Selected IIH women, small sample size |
|  | Edwards et al. 2013 | 17 (1/16) | Revised Friedman | 53 (15/38) | - | MS, INC, FC | CSF | ELISA | NS (P>0.05) | - | - |
|  | Pandit et al. 2024 | 13 (2/11) | Modified Dandy | 20 (14/6) | - | Heterogeneous patients | CSF | SWATH-MS proteomics | IIH>controls (P=0.01) | - | Lack of absolute quantification data, lack of a validation cohort, fewer women in control group |
| **Sortilin-1/Lipocalin-2/Autotaxin/Decorin** | Dündar et al. 2022 | 24 (10/14) | Dandy | 21 (11/10) | Age, sex, BMI | HC | CSF | ELISA | Sortilin-1/Lipocalin-2/Autotaxin: IIH>HC (P<0.001)  Decorin: IIH<HC (P=0.008) | No correlation with CSF pressure | Not tested in serum, the biochemical properties not included |
| **Inflammatory proteins^*^** | Brettschneider et al. 2011 | 18 (4/14) | Modified Dandy | 18 (4/14) | Age, sex | TTH | CSF | 2-D DIGE | IIH>controls (P<0.05) | - | Not strictly normal controls |
| **Section II. Disease activity and organ injury markers** | | | | | | | | | | | |
| **NSE** | Samancı et al. 2017 | 36 (4/32) | Revised Friedman | 40 (4/36) | Age, sex, BMI | HC | Serum | ELISA | IIH>HC (P=0.824) | No correlations with visual loss, relapse or PE | Small sample size |
| **OCBs** | Altıokka-Uzun et al. 2015 | 26 (1/25) | Revised Friedman | - | - | - | Serum and CSF | IEF, immunoblotting | Positive in 8/26 (30.77%) patients | Associated with vision loss | Small series from a single center |
|  | Altıokka-Uzun et al. 2017 | 27 | Revised Friedman | - | - | - | Serum and CSF | IEF, immunoblotting | Positive in 9/27 (33.3%) patients | - | - |
|  | El-Tamawy et al. 2019 | 27 (0/27) | Modified Dandy | - | - | - | CSF | IEF, immunoblotting | Positive in 22.2% patients | - | - |
| **IgG synthesis** | Inshasi et al. 1995 | 11 (2/9) | Modified Dandy | - | - | - | Serum and CSF | Single immunoelectrophoresis, IEF | Local IgG synthesis was positive, while qualitative measurement was negative. | - | - |
| **IgG binding patterns** | Ekizoglu et al. 2012 | 29 (3/26) | Modified Dandy | 283 | - | Encephalitis | Serum | IHC | NS (P>0.05) | - | - |
| **GFAP** | Engel et al. 2023 | 87 (12/75) | Revised Friedman | 114 | Age, sex | MS, HC | Serum and CSF | SiMoA | Serum: NS (P>0.05)  CSF: IIH>HC (P<0.01) | - | - |
| **anti-GFAP antibodies** | Yetimler et al. 2021 | 58 (6/52) | Revised Friedman | - | - | - | Serum | CBA, IHC | Found in 2 IIH patients | - | - |
| **NfL** | Beier et al. 2020 | 35 (0/35) | Friedman | - | - | - | CSF | SiMoA | IIH patients: moderate and severe PE> patients with minor and no PE (P<0.001) | Associated with CSF pressure, bilateral visual field defects and bilateral optic nerve atrophy | Timing of CSF sampling |
|  | Knoche et al. 2023 | 35 (9/26) | Revised Friedman | 12 (4/8) | - | HC | CSF | - | IIH>HC, IIH patients: moderate or sereve PE>wild or no PE (both P<0.05) | Associated with the severity of optic nerve damage, PE and CSF pressure | Small cohort, its retrospective nature, selection bias |
|  | Engel et al. 2023 | 87 (12/75) | Revised Friedman | 114 | Age, sex | MS, PNP, HC | Serum and CSF | SiMoA | Serum: IIH<MS, IIH<PNP  CSF: IIH>HC (all P<0.05) | Associated with CSF pressure | Testing method |
|  | Svart et al. 2024 | 37 (1/36) | Revised Friedman | 35 (1/34) | Age, sex, BMI | HC | CSF and plasma | SiMoA | CSF: IIH>HC, IIH patients: severe PE>mild-moderate PE  Plasma: IIH>HC (all P<0.05) | Associated with PE, visual field defects and CSF pressure | Testing sample |
| **Section III. Mechanistic inflammatory markers** | | | | | | | | | | | |
| **Interleukins** | | | | | | | | | | | |
| **IL-1α** | Dhungana et al. 2009 | 8 | Modified Dandy | 8 | - | MS, migraine, TTH | Serum and CSF | Cytokine antibody arrays, ELISA | Serum: IIH>controls (P<0.05)  CSF: NS (P>0.05) | - | - |
| **IL-1β** | Dhungana et al. 2009 | 26 (0/26) | Modified Dandy | 26 (0/26) | - | Heterogeneous patients | Serum | MIA | NS (P>0.05) | - | - |
|  | Edwards et al. 2013 | 7 | Revised Friedman | 26 | - | MS, INC | Serum | MIA | NS (P>0.05) | - | - |
|  | Edwards et al. 2013 | 17 (1/16) | Revised Friedman | 53 (15/38) | - | MS, INC, FC | CSF | MIA | NS (P>0.05) | - | - |
|  | Samancı et al. 2017 | 36 (4/32) | Revised Friedman | 40 (4/36) | Age, sex, BMI | HC | Serum | MIA | IIH>HC (P=0.012) | Associated with IIH relapse, no correlations with disease duration or visual impairment | IIH cases in remission, CSF not obtained in HC |
| **IL-2** | Hannerz et al. 2011 | 12 (0/12) | ICHD-II | 12 (0/12) | Age, sex | HC | CSF and PBMC | - | IIH> HC (P>0.05) | - | Small group sizes, rigorous inclusion criteria |
|  | Edwards et al. 2013 | 17 (1/16) | Revised Friedman | 53 (15/38) | - | MS, INC, FC | CSF | MIA | IIH>MS (P<0.001) | - | - |
| **IL-4** | Edwards et al. 2010 | 11 | - | 35 | - | CIDP, CIS, MS | CSF | MIA | NS (P=0.38) | - | - |
|  | Edwards et al. 2013 | 7 | Revised Friedman | 26 | - | MS, INC | Serum | MIA | NS (P>0.05) | - | - |
|  | Edwards et al. 2013 | 17 (1/16) | Revised Friedman | 53 (15/38) | - | MS, INC, FC | CSF | MIA | IIH>MS (P<0.001) | - | - |
|  | Altıokka-Uzun et al. 2015 | 26 (1/25) | Revised Friedman | 33 | Age, sex | RRMS, HC | Serum and CSF | ELISA | Serum: IIH>RRMS, IIH>HC  CSF: IIH>RRMS (all P<0.05) | - | Small series from a single center |
|  | El-Tamawy et al. 2019 | 27 (0/27) | Modified Dandy | 21 (0/21) | Age, sex | HC | Serum | ELISA | IIH> HC (P<0.001) | - | - |
|  | Genizi et al. 2023 | 43 (20/23) | Revised Friedman | 17 (7/10) | - | Non-IIH patients | CSF | ELISA | NS (P>0.05) | - | Small control group, not measure serum levels |
| **IL-6** | Reihani-Kermani et al. 2008 | 14 (0/14) | Modified Dandy | 14 (0/14) | - | Non-IIH patients | CSF | ELISA | IIH>controls (P<0.05) | - | - |
|  | Ball et al. 2009 | 26 (0/26) | Modified Dandy | 62 (22/40) | Age, sex, BMI | Heterogeneous patients | CSF | MIA | NS (P>0.05) | - | - |
|  | Hannerz et al. 2011 | 12 (0/12) | ICHD-II | 12 (0/12) | Age, sex | HC | CSF and PBMC | - | CSF: IIH>HC (P=0.044)  PBMC: IIH>HC (P>0.05) | Associated with CSF pressure | Small group sizes, rigorous inclusion criteria |
|  | Edwards et al. 2013 | 17 (1/16) | Revised Friedman | 53 (15/38) | - | MS, INC, FC | CSF | MIA | NS (P>0.05) | - | - |
|  | Edwards et al. 2013 | 7 | Revised Friedman | 26 | - | MS, INC | Serum | MIA | NS (P>0.05) | - | - |
|  | Samancı et al. 2017 | 36 (4/32) | Revised Friedman | 40 (4/36) | Age, sex, BMI | HC | Serum | MIA | NS (P=0.491) | No correlations with disease duration or visual impairment | IIH cases in remission, CSF not obtained in HC |
|  | Karabork et al. 2024 | 16 (2/14) | Revised Friedman | 22 (6/16) | - | MS | CSF | ELISA | NS (P=0.064) | - | Small sample size, not measure serum levels, testing methods, CSF not obtained in HC |
| **IL-8** | Ball et al. 2009 | 26 (0/26) | Modified Dandy | 62 (22/40) | Age, sex, BMI | Heterogeneous patients | Serum and CSF | MIA | Serum: IIH<controls (P=0.008)  CSF: NS (P>0.05) | - | - |
|  | Edwards et al. 2013 | 7 | Revised Friedman | 26 | - | MS, INC | Serum | MIA | NS (P>0.05) | - | - |
|  | Edwards et al. 2013 | 17 (1/16) | Revised Friedman | 53 (15/38) | - | MS, INC, FC | CSF | MIA | NS (P>0.05) | - | - |
|  | Samancı et al. 2017 | 36 (4/32) | Revised Friedman | 40 (4/36) | Age, sex, BMI | HC | Serum | MIA | IIH<HC (P<0.001) | Associated with IIH relapse, no correlations with disease duration or visual impairment | IIH cases in remission, CSF not obtained in HC |
| **IL-10** | Reihani-Kermani et al. 2008 | 14 (0/14) | Modified Dandy | 14 (0/14) | - | Non-IIH patients | CSF | ELISA | NS (P>0.05) | - | - |
|  | Edwards et al. 2010 | 11 | - | 35 | - | CIDP, CIS, MS | CSF | MIA | IIH<MS, IIH<CIS  (both P<0.05) | - | - |
|  | Hannerz et al. 2011 | 12 (0/12) | ICHD-II | 12 (0/12) | Age, sex | HC | CSF and PBMC | - | IIH<HC (P>0.05) | - | Small group sizes, rigorous inclusion criteria |
|  | Edwards et al. 2013 | 17 (1/16) | Revised Friedman | 53 (15/38) | - | MS, INC, FC | CSF | MIA | IIH<INC (P=0.0043) | - | - |
|  | Edwards et al. 2013 | 7 | Revised Friedman | 26 | - | MS, INC | Serum | MIA | NS (P>0.05) | - | - |
|  | Altıokka-Uzun et al. 2015 | 26 (1/25) | Revised Friedman | 33 | Age, sex | RRMS, HC | Serum and CSF | ELISA | Serum: IIH>RRMS, IIH>HC  CSF: IIH>RRMS (all P<0.05) | - | Small series from a single center |
|  | El-Tamawy et al. 2019 | 27 (0/27) | Modified Dandy | 21 (0/21) | Age, sex | HC | Serum | ELISA | IIH> HC (P<0.001) | - | - |
|  | Genizi et al. 2023 | 43 (20/23) | Revised Friedman | 17 (7/10) | - | Non-IIH patients | CSF | ELISA | NS (P>0.05) | - | Small control group, not measure serum levels |
| **IL-12** | Altıokka-Uzun et al. 2015 | 26 (1/25) | Revised Friedman | 33 | Age, sex | RRMS, HC | Serum and CSF | ELISA | Serum: IIH>RRMS, IIH>HC  CSF: IIH>RRMS (all P<0.05) | - | Small series from a single center |
| **IL-12p70** | Edwards et al. 2013 | 17 (1/16) | Revised Friedman | 53 (15/38) | - | MS, INC, FC | Serum and CSF | MIA | NS (P>0.05) | - | - |
| **IL-17** | Edwards et al. 2010 | 11 | - | 35 | - | CIDP, CIS, MS | CSF | MIA | IIH>CIDP, IIH>CIS  (both P<0.05) | - | - |
|  | Edwards et al. 2013 | 17 (1/16) | Revised Friedman | 53 (15/38) | - | MS, INC, FC | CSF | MIA | IIH>MS; IIH>FC  (both P<0.01) | - | - |
|  | Altıokka-Uzun et al. 2015 | 26 (1/25) | Revised Friedman | 33 | Age, sex | RRMS, HC | Serum and CSF | ELISA | Serum: IIH>RRMS, IIH>HC  CSF: IIH>RRMS (all P<0.05) | - | Small series from a single center |
|  | Genizi et al. 2023 | 43 (20/23) | Revised Friedman | 17 (7/10) | - | Non-IIH patients | CSF | ELISA | NS (P>0.05) | - | Small control group, not measure serum levels |
| **IL-22** | Edwards et al. 2013 | 17 (1/16) | Revised Friedman | 53 (15/38) | - | MS, INC, FC | Serum and CSF | ELISA | NS (P>0.05) | - | - |
| **IL-23** | Hannerz et al. 2011 | 12 (0/12) | ICHD-II | 12 (0/12) | Age, sex | HC | CSF and PBMC | - | CSF: IIH>HC (P=0.001)  PBMC: IIH>HC (P>0.05) | - | Small group sizes, rigorous inclusion criteria |
| **IL-33** | Dündar et al. 2022 | 24 (10/14) | Dandy | 21 (11/10) | Age, sex, BMI | HC | CSF | ELISA | IIH>HC (P<0.001) | No correlation with CSF pressure | Not tested in serum, the biochemical properties not included |
| **Chemokines** | | | | | | | | | | | |
| **CCL2** | Dhungana et al. 2009 | 8 | Modified Dandy | 8 | - | MS, migraine, TTH | Serum and CSF | Cytokine antibody arrays, ELISA | Serum: NS (P>0.05)  CSF: IIH>controls (P<0.05) | No correlation with CSF pressure | - |
|  | Ball et al. 2009 | 26 (0/26) | Modified Dandy | 62 (22/40) | Age, sex, BMI | Heterogeneous patients | Serum and CSF | MIA | Serum: IIH<controls (P=0.04)  CSF: NS (P>0.05) | - | - |
|  | Samancı et al. 2017 | 36 (4/32) | Revised Friedman | 40 (4/36) | Age, sex, BMI | HC | Serum | MIA | NS (P=0.429) | No correlations with disease duration or visual impairment | IIH cases in remission, CSF not obtained in HC |
|  | Genizi et al. 2023 | 43 (20/23) | Revised Friedman | 17 (7/10) | - | Non-IIH patients | CSF | ELISA | IIH>controls (P<0.03) | - | Small control group, not measure serum levels, testing methods |
| **CCL7/CCL8** | Dhungana et al. 2009 | 8 | Modified Dandy | 8 | - | MS, migraine, TTH | Serum | Cytokine antibody arrays | IIH>controls (P<0.05) | - | - |
|  | Genizi et al. 2023 | 43 (20/23) | Revised Friedman | 17 (7/10) | - | Non-IIH patients | CSF | ELISA | NS (P>0.05) | - | Small control group, not measure serum levels |
| **CCL13** | Genizi et al. 2023 | 43 (20/23) | Revised Friedman | 17 (7/10) | - | Non-IIH patients | CSF | ELISA | NS (P>0.05) | - | Small control group, not measure serum levels |
| **CXCL10** | Dhungana et al. 2009 | 26 (0/26) | Modified Dandy | 26 (0/26) | - | Heterogeneous patients | Serum and CSF | MIA | NS (P>0.05) | - | - |
| **TNF-α** | Ball et al. 2009 | 26 (0/26) | Modified Dandy | 62 (22/40) | Age, sex, BMI | Heterogeneous patients | Serum | MIA | NS (P>0.05) | - | - |
|  | Hannerz et al. 2011 | 12 (0/12) | ICHD-II | 12 (0/12) | Age, sex | HC | CSF and PBMC | - | IIH>HC (P>0.05) | - | Small group sizes, rigorous inclusion criteria |
|  | Edwards et al. 2013 | 7 | Revised Friedman | 26 | - | MS, INC | Serum | MIA | NS (P>0.05) | - | - |
|  | Edwards et al. 2013 | 17 (1/16) | Revised Friedman | 53 (15/38) | - | MS, INC, FC | CSF | MIA | NS (P>0.05) | - | - |
|  | Altıokka-Uzun et al. 2015 | 26 (1/25) | Revised Friedman | 33 | Age, sex | RRMS, HC | Serum | ELISA | IIH>RRMS, IIH>HC  (both P<0.05) | - | Small series from a single center |
|  | Samancı et al. 2017 | 36 (4/32) | Revised Friedman | 40 (4/36) | Age, sex, BMI | HC | Serum | MIA | IIH<HC (P=0.008) | Associated with IIH relapse, no correlations with disease duration or visual impairment | IIH cases in remission, CSF not obtained in HC |
|  | El-Tamawy et al. 2019 | 27 (0/27) | Modified Dandy | 21 (0/21) | Age, sex | HC | Serum | ELISA | IIH> HC (P<0.001) | Positive correlation with CSF pressure | - |
|  | Fahmy et al. 2021 | 36 (1/35) | ICHD-III | 30 (1/29) | Age, sex, BMI | HC | Serum | ELISA | IIH>HC (P<0.001) | Associated with grade of perimetry and CSF pressure, no correlations with disease duration, headache severity, relapse rate, visual acuity, or PE, a significant predictor of the severity of the visual field affection | Different disease stages |
|  | Karabork et al. 2024 | 16 (2/14) | Revised Friedman | 22 (6/16) | - | MS | CSF | ELISA | NS (P=0.089) | - | Small sample size, not measure serum levels, testing methods, CSF not obtained in HC |
| **IFN-γ** | Edwards et al. 2010 | 11 | - | 35 | - | CIDP, CIS, MS | CSF | MIA | NS (P=0.5) | - | - |
|  | Edwards et al. 2013 | 7 | Revised Friedman | 26 | - | MS, INC | Serum | MIA | NS (P>0.05) | - | - |
|  | Edwards et al. 2013 | 17 (1/16) | Revised Friedman | 53 (15/38) | - | MS, INC, FC | CSF | MIA | IIH>MS (P<0.0001) | - | - |
|  | Altıokka-Uzun et al. 2015 | 26 (1/25) | Revised Friedman | 33 | Age, sex | RRMS, HC | Serum and CSF | ELISA | Serum: IIH>RRMS, IIH>HC  CSF: IIH>RRMS (all P<0.05) | - | Small series from a single center |
|  | Genizi et al. 2023 | 43 (20/23) | Revised Friedman | 17 (7/10) | - | Non-IIH patients | CSF | ELISA | NS (P>0.05) | - | Small control group, not measure serum levels |
| **TGF-β** | Edwards et al. 2013 | 17 (1/16) | Revised Friedman | 53 (15/38) | - | MS, INC, FC | CSF | ELISA | NS (P>0.05) | - | - |

**Abbreviations:** 2-D DIGE, two-dimensional fluorescence differential in-gel electrophoresis; BCVA, best‑corrected visual acuity; BMI, body mass index; CBA, cell-based assay; CCL, C-C motif chemokine ligand; CIDP, chronic inflammatory demyelinating polyneuropathy; CIS, clinically isolated syndrome; CRP, C-reactive protein; CSF, cerebrospinal fluid; CXCL, C-X-C motif chemokine ligand; ELISA, enzyme-linked immunosorbent assay; F, female; FC, functional conditions; GFAP, glial fibrillary acidic protein; HC, healthy controls; hs-CRP, high-sensitivity C-reactive protein; ICHD-II, The International Classification of Headache Disorders: 2nd edition; ICHD-III, International Classification of Headache Disorders, 3rd edition; IEF, isoelectric focusing; IFN, interferon; Ig, immunoglobulin; IHC, immunohistochemistry; IIH, idiopathic intracranial hypertension; IL, interleukin; INC, inflammatory neurological conditions; LC-MS, liquid chromatography-mass spectrometry; M, male; MIA, multiplex immunoassay; MRV, magnetic resonance venography; MS, multiple sclerosis; NfL, neurofilament light chain; NLR, neutrophil-to-lymphocyte ratio; NS, not significant; NSE, neuron-specific enolase; OCB, oligoclonal band; PBMC, peripheral blood mononuclear cell; PE, papilledema; PLR, platelet-to-lymphocyte ratio; PNP, polyneuropathy; RNFLT, retinal nerve fiber layer thickness; RRMS, relapsing-remitting multiple sclerosis; SiMoA, single molecule array; SWATH-MS, sequential window acquisition of all theoretical fragment ion spectra-mass spectrometry; TGF, transforming growth factor; TNF, tumor necrosis factor; TTH, tension-type headache.

^#^ include (i) signs and symptoms of increased intracranial pressure; (ii) no localising signs except abducens nerve palsy; (iii) IIH diagnosed by continuous intracranial pressure monitoring using an epidural sensor; (iv) CSF with normal chemical composition; and (v) normal neuroimaging.

^*^ include immunoglobulin heavy constant alpha 1, alpha-1-antitrypsin, serotransferrin, and haptoglobin.
